# Global Policy and Practice for Intrauterine Fetal Resuscitation During Fetal Surgery for Open Spina Bifida Repair

**DOI:** 10.1001/jamanetworkopen.2023.9855

**Published:** 2023-04-25

**Authors:** Katie Gallagher, Neeltje Crombag, Kavita Prashar, Jan Deprest, Sebastien Ourselin, Anna L. David, Neil Marlow

**Affiliations:** 1 UCL Elizabeth Garrett Anderson Institute for Women's Health, University College London, London, United Kingdom; 2 Department of Obstetrics and Gynaecology, Fetal Medicine Unit, University Hospitals KU Leuven, Leuven, Belgium; 3 School of Biomedical Engineering and Imaging Sciences, King’s College London, London, United Kingdom

## Abstract

**Importance:**

Globally accepted recommendations suggest that a woman should be between 19 weeks and 25 weeks plus 6 days of pregnancy to be considered eligible for fetal closure of open spina bifida. A fetus requiring emergency delivery during surgery is therefore potentially considered viable and thus eligible for resuscitation. There is little evidence, however, to support how this scenario is addressed in clinical practice.

**Objective:**

To explore current policy and practice for fetal resuscitation during fetal surgery for open spina bifida in centers undertaking fetal surgery.

**Design, Setting, and Participants:**

An online survey was designed to identify current policies and practices in place to support fetal surgery for open spina bifida, exploring experiences and management of emergency fetal delivery and fetal death during surgery. The survey was emailed to 47 fetal surgery centers in 11 countries where fetal spina bifida repair is currently performed. These centers were identified through the literature, the International Society for Prenatal Diagnosis center repository, and an internet search. Centers were contacted between January 15 and May 31, 2021. Individuals volunteered participation through choosing to complete the survey.

**Main Outcomes and Measures:**

The survey comprised 33 questions of mixed multiple choice, option selection, and open-ended formats. Questions explored policy and practice supporting fetal and neonatal resuscitation during fetal surgery for open spina bifida.

**Results:**

Responses were obtained from 28 of 47 centers (60%) in 11 countries. Twenty cases of fetal resuscitation during fetal surgery during the last 5 years were reported across 10 centers. Four cases of emergency delivery during fetal surgery after maternal and/or fetal complications during the last 5 years were reported across 3 centers. Fewer than half the 28 centers (n = 12 [43%]) had policies in place to support practice in the event of either imminent fetal death (during or after fetal surgery) or the need for emergency fetal delivery during fetal surgery. Twenty of 24 centers (83%) reported preoperative parental counseling on the potential need for fetal resuscitation prior to fetal surgery. The gestational age at which centers would attempt neonatal resuscitation after emergency delivery varied from 22 weeks and 0 days to more than 28 weeks.

**Conclusions:**

In this global survey study of 28 fetal surgical centers, there was no standard practice about how fetal resuscitation or subsequent neonatal resuscitation was managed during open spina bifida repair. Further collaboration between professionals and parents is required to ensure sharing of information to support knowledge development in this area.

## Key Points

**Question:**

What is current global practice for fetal resuscitation, emergency delivery, and subsequent neonatal resuscitation during fetal surgery for open spina bifida repair?

**Findings:**

This global survey study of 28 centers in 11 countries performing fetal surgery for open spina bifida repair identified variability in fetal resuscitation and subsequent neonatal resuscitation practices. The gestational age at which neonatal resuscitation would be attempted after emergency delivery varied from 22 weeks and 0 days to more than 28 weeks.

**Meaning:**

This study suggests that further collaborative work between centers is required to assess how decisions are made about fetal resuscitation during fetal surgery for open spina bifida repair and the effect of those decisions on professionals and parents.

## Teaser

This survey study assesses current policy and practice for fetal resuscitation during fetal surgery for open spina bifida in centers undertaking fetal surgery.

## Introduction

Advances in preclinical research, fetal imaging, surgical technology, and clinician skill and experience mean that fetal surgery has become an acceptable form of fetal therapy for an increasing number of antenatally diagnosed fetal anomalies.^1,2^ Open spina bifida is one example of a fetal anomaly that can be treated through antenatal repair. It is the most common neural tube defect that can be diagnosed during pregnancy, occurring in 4.72 per 10 000 births in the European Union in 2017.^3^ The most prevalent forms of open spina bifida that can be treated through fetal therapy are myelomeningocele and myeloschisis; in myelomeningocele, the spinal cord extrudes into a sac filled with cerebrospinal fluid, whereas in myeloschisis, the neural tissue does not extrude and is level with the surrounding skin.^4,5^ Although open spina bifida is not lethal, it is not curable through surgery, and long-term effects can include paralysis, cognitive disabilities, and hydrocephalus, along with social and emotional issues.^6,7^

There is level I evidence that prenatal open spina bifida repair by hysterotomy (open repair) improves outcomes.^6^ Ongoing efforts are being made to reduce the invasiveness of the operation by prenatal repair either by mini-hysterotomy^8^ or fetoscopy.^9^ A significant risk during either surgery, however, is fetal compromise, potentially leading to fetal demise. If fetal and/or maternal complications develop, in utero fetal resuscitation and/or emergency delivery and subsequent neonatal resuscitation may be required to improve fetal or neonatal survival and/or reduce maternal adverse outcomes.^10^ A recent review of outcomes of 300 children undergoing intended fetoscopic open spina bifida repair found that the surgery was not completed in 9 cases: 1 child was delivered intraoperatively for bradycardia unresponsive to in utero resuscitation and 8 cases were abandoned due to fetal heart rate abnormalities (bradycardia) at different time points, with 1 of these cases requiring immediate cesarean delivery.^9^

Globally accepted evidence-based practice for fetal closure of open spina bifida recommends that a woman should be between 19 weeks and 25 weeks plus 6 days of pregnancy to be eligible for fetal surgery, with only a few centers performing surgery beyond this date and up to 31 weeks of gestation.^4,11^ A fetus requiring emergency delivery during surgery is therefore considered viable and thus eligible for resuscitation; however, clinical practice is apparently geographically dependent and may vary according to legal definitions in the relevant country.^12^ In the UK and US, guidelines recommend that active resuscitation is considered from 22 weeks of gestation if the neonate displays signs of life.^13,14^ There is wide variation across Europe, and prior to 26 weeks of gestation, treatment may be active, minimal interventions only, palliative, or requiring parental consent.^13,15^ It is unclear whether neonatal resuscitation guidelines in each country apply to infants with a serious congenital anomaly, who, in addition, experience serious fetal distress and eventually are delivered alive after a decision by the clinical team to expedite delivery during fetal surgery. It is also unknown how this scenario is addressed by parents who travel across country borders for fetal surgery. This study therefore aims to assess current policy and practice for both fetal resuscitation during fetal surgery and neonatal resuscitation after emergency delivery during fetal surgery, in fetal centers undertaking this surgery across the world.

## Methods

We designed a survey to capture global policy and practice supporting fetal and, where applicable, neonatal resuscitation during fetal surgery for open spina bifida. The survey, developed through literature review, comprised 33 questions of mixed multiple-choice option selections and open-ended formats (eAppendix in Supplement 1). To improve the usability of the survey, it was divided into 4 sections, each with its own brief introduction explaining the purpose and rationale behind the questions: (1) fetal surgery center information, (2) in utero fetal resuscitation, (3) emergency delivery during surgery and subsequent neonatal resuscitation, and (4) neonatal palliative care. There was a final open-ended question for participants to add any further comments. The survey was piloted and peer reviewed by experts in the field of fetal medicine, fetal surgery, and neonatology to ensure content validity. The study concept and results were also reviewed by the Public and Patient Involvement Advisory Group for the GIFT-Surg (Guided Instrumentation for Fetal Therapy and Surgery) study, the overarching project aiming to improve surgical techniques and explore the acceptability of fetal surgery.^16^ The survey was hosted by Opinio 7.19, a UCL web-based survey tool (ObjectPlanet Inc). The participant information sheet and digital consent form were included at the start of the survey, for ease of completion. The study was reviewed and approved by the University College London Research Ethics Committee and is reported in line with the American Association for Public Opinion Research (AAPOR) reporting guideline.

### Data Collection

We identified 47 eligible fetal surgery centers through previous research exploring the global availability of fetal surgery for open spina bifida, contact details for which are available on the International Society for Prenatal Diagnosis website, through literature review and internet search for additional centers now performing this surgery, and through recommendation from fellow fetal surgeons.^11,17^ An email was sent to all contacts providing information about the study aims and inviting participation, with a link to the survey. A follow-up email was sent to all potential participants after 2 weeks to encourage participation and to thank those who had already taken part. Data were collected between January 15 and May 31, 2021.

### Participants

Centers were eligible for inclusion if they were currently performing, or had previously undertaken, fetal surgery for open spina bifida repair. Individuals at each center were eligible to complete the survey if they were involved in the organization or conduct of fetal surgery for open spina bifida, including but not limited to maternal-fetal specialists, fetal surgeons, neurosurgeons, neonatologists, midwives, and fetal center coordinators. As participants were contacted individually, responses were limited to 1 survey per center.

### Statistical Analysis

Data were downloaded from Opinio and imported into Microsoft Excel, version 1808 (Microsoft Corp), to facilitate data analysis. Descriptive statistics explored differences in approaches to clinical practice, frequency of policies in place to support fetal resuscitation (both in utero and emergency delivery), and approaches toward parental counseling and managing fetal or neonatal death. Content analysis evaluated responses to open-ended questions, exploring similarities and differences between participants.^18^

## Results

From the 47 eligible centers currently performing fetal surgery for open spina bifida, we received responses from 28 centers (response rate, 60%). Most centers were in North America (n = 13), followed by Europe (n = 7), South America (n = 5), and Asia (n = 3). Not all respondents completed all questions; we included all surveys, and adjusted response rates depending on the number of respondents per question.

### Overall Findings

Most individual participants identified as being fetal medicine specialists (75% [21 of 28]). Twelve centers (43%) offered open fetal surgery only, 4 (14%) offered fetoscopic fetal surgery only, and 12 (43%) offered both modalities. Of the 24 centers performing open fetal surgery, the median number of cases per year was 8 (range, 1-20). Of 16 centers performing fetoscopic fetal surgery, 15 provided information; the median number of fetal surgical procedures performed per year was 10 (total number, 144; range, 2-30).

All centers were asked about cases requiring fetal resuscitation during fetal surgery in the past 5 years; 20 cases of fetal resuscitation were reported across 10 centers. Free text information provided insight into measures taken to address signs of fetal cardiac compromise, including fetal administration of bicarbonate, atropine, and/or epinephrine; uterine or fetal repositioning; pausing procedures; and warm saline uterine infusions.

### Policy and Practices

Fifteen centers responded to the question exploring fetal resuscitation policy; 11 (73%) had a specific policy. Twenty-four centers responded to questions about parental counseling about potential fetal complications during fetal surgery. Twenty (83%) reported that parents were counseled on the potential need for fetal resuscitation, with counseling taking place both during the initial consultation (n = 14 [58%]) and/or immediately prior to fetal surgery (n = 20 [83%]). Counseling was undertaken by a range of professionals including maternal-fetal medicine specialists, neonatologists, cardiologists, anesthesiologists, and fetal surgeons. Twenty-four centers responded to questions about information sharing among health care professionals prior to fetal surgery, and 19 centers (79%) indicated that fetal resuscitation measures were discussed with the whole surgical team immediately prior to fetal surgery. Parent or patient representative involvement in the development of fetal surgery resuscitation policy was reported by 3 centers; fetal surgeons (n = 16), fetal medicine specialists (n = 15), and anesthesiologists (n = 13) had the greatest input.

### Emergency Delivery During Fetal Surgery and Subsequent Neonatal Resuscitation

Centers were asked whether in the past 5 years they had experienced any instances of emergency delivery during fetal surgery for open spina bifida. Three of 20 centers (15%) reported 4 cases in which emergency delivery had been necessary (2 each during open or fetoscopic surgery) at between 23 and 26 weeks of gestation. Outcomes were reported by only 1 center; the infant died during attempted resuscitation efforts at 23 weeks of gestation. Twelve of 23 centers had a specific policy for emergency delivery during fetal surgery, and 8 had a policy for subsequent neonatal resuscitation; 20 centers reported that parents were counseled on potential delivery during fetal surgery, either prior to fetal surgery at the initial consultation (n = 12) and/or again when written consent for fetal surgery was being sought (n = 18).

When asked at what gestational age centers would consider neonatal resuscitation in the event of fetal delivery, 23 centers responded, indicating a wide range from 22 weeks to more than 28 weeks of gestation, varying both within and between countries (Box). Signed parental consent was gained for fetal delivery and subsequent neonatal resuscitation in 11 and 8 of these 24 centers, respectively.

Box. **Gestational Age at Which Neonatal Resuscitation Would Be Considered by Country of Responding Center Performing Fetal Surgery for Open Spina Bifida Repair**

**When neonatal resuscitation would be considered (weeks of gestation plus days)**

**22 + 0 to 22 + 6**

US (2 responses)

**23 + 0 to 23 + 6**

US (2 responses)
Germany

**24 + 0 to 24 + 6**

Canada
France
Argentina
Germany
US (5 responses)
Taiwan

**25 + 0 to 25 + 6**

US (1 response)
Chile

**26 + 0 to 26 + 6**

Columbia
Peru (2 responses)
UK

**28 + 0 to 28 + 6**

Iran

**Other—“Wouldn’t deliver after 28 weeks for MMC but wouldn’t operate after 26 weeks”**

France

Abbreviation: MMC, myelomeningocele.

### Fetal and Neonatal Palliative and End-of-Life Care

Twelve of 22 responding centers reported that a plan for neonatal palliative care was discussed with parents in the case of neonatal resuscitation after fetal delivery, indicating that neonatologists predominately led these discussions. Of these centers, 12 had policies in place to support practice in the case of fetal death, during fetal surgery or after fetal delivery, while 10 did not. Free text comments highlighted the areas of support available to parents in this scenario, including being able to see their infant as soon as the anesthesia was reversed and the mother was awake or they were ready (n = 3) and providing referrals for ongoing professional support (n = 4). Local palliative or hospice teams would be available to support families in 13 of 22 responding centers.

## Discussion

The aim of this survey was to make an inventory of current policies and practices for fetal resuscitation during fetal surgery for open spina bifida, emergency delivery, and subsequent neonatal resuscitation, in centers where this surgery is performed. There is variability in many areas of practice, including policy provision, whether parental consent is sought, the gestational age from which neonatal resuscitation would be initiated, and management of fetal or neonatal death. We have collated previously unknown information around multiple aspects of fetal surgery for open spina bifida from 60% of all identified surgical centers undertaking this surgery, including reported cases of resuscitation, approaches to parental engagement and counseling, and palliative care.

In areas of practice such as rare diseases, or where new procedures are introduced, policy dictating evidence-based discussions is vital to ensure that the rights of patients are upheld and that patients have the latest information about the procedure to facilitate autonomous decision-making and informed consent.^19^ These discussions with patients require consideration of potential complications and the actions to be undertaken should these complications ensue. Our findings highlight that a number of fetal surgical centers, however, have no such policy in place and, therefore, may not discuss in advance their response to these rare but critical events with the pregnant women, their partners, and their families. Although instances of fetal resuscitation or emergency delivery may be rare, they are potentially catastrophic for the woman and her family. Coordinated effort is therefore imperative from fetal surgical centers in making progress in developing appropriate policy^20^ and ensuring that clinical decisions are planned, and not reactive, during fetal surgery. These decisions should be underpinned by discussions that support informed decision-making with families, to minimize any potential confusion, distress, or surprise resulting from actions undertaken in the event of surgical complications, including fetal delivery and subsequent neonatal resuscitation.

To support the development and implementation of policy, efforts should be made to use the wealth of experience of patients and their families who have invested significant time and emotion into fetal surgery, to ensure that practice remains patient centered while meeting the broader needs of families.^21^ Our survey highlighted that only 3 centers reported patient representative engagement in development of their fetal surgery policy. Wider evidence has shown that patients receiving rare or emerging treatment often feel empowered to engage in patient advocacy work to support the improvement of policy, research, patient information, and education and can be strong influences for change.^20^ Engaging patient representatives in the process of policy development is therefore vital in determining what information should be shared, and how, to ensure optimal care of patients and their families and determine best practice in areas of limited experience.

Extrapolating best practice, however, is complicated through the cultural setting in which fetal surgery is taking place.^22^ Our findings highlight wide variability in the gestational age at which neonatal resuscitation would be initiated after emergency delivery, not only between but within countries, reflecting previous research from neonatal settings and indicating different national policies and attitudes toward neonatal resuscitation.^23^ Limited literature around neonatal resuscitation after fetal delivery, with the additional complication of potentially partially completed fetal surgery, makes it difficult to determine whether neonatal guidelines are applicable in this setting, or whether, as some centers highlighted in free text responses, a more individualized approach should be followed to determine infant viability and eligibility for resuscitation. Moving away from gestational age and toward an individualized model of neonatal resuscitation requires detailed discussions with parents prior to fetal surgery to understand their experiences, emotions, and decision-making preferences for their infant.^24^ Increased multidisciplinary engagement in developing fetal surgical policies may support the implementation of these discussions; our survey highlighted the minimal role of neonatologists currently involved in policy development in comparison with fetal surgeons, fetal medicine specialists, and anesthesiologists. Engaging neonatologists in this process may facilitate informed decision-making for parents prior to fetal surgery.

Currently, the country in which a mother undergoes fetal surgery, along with the attitude of clinicians toward any national guidelines, appears to influence whether an infant would potentially be resuscitated after fetal delivery. This is particularly important when parents consider where to travel for fetal surgery. The short time between determining eligibility for surgery, the gestational age at which surgery can be performed, and that surgery is performed only by experienced teams in certain maternal-fetal medicine centers, mean that parents and professionals may travel to different centers or countries for this procedure.^25^ This may result in parental and professional confusion around guidelines and expectations in the event of emergency delivery during fetal surgery. Further research is required to assess how professionals make clinical decisions around resuscitation in this scenario and the role of parents in this decision-making process.

Finally, our findings highlighted limited center policy to guide parents or professionals in the event of fetal or neonatal death, or in the parallel planning of neonatal palliative care. Neonatal palliative care provides support to parents to plan for improving their infant’s quality of life when prolongation of life is no longer the aim of care, or for when their prognosis is uncertain.^26^ Arguably, for infants with a diagnosis of open spina bifida whose families have opted for surgical intervention, and who are therefore at risk for preterm delivery with potentially complex comorbidities, a palliative care consultation could be warranted to explore the values of the families, the risks to the infants of preterm delivery, and what options parents may have.^27^ A parallel planning approach may facilitate the development of center policy to support parents and professionals in the event of fetal death, ensuring equity of service provision and facilitating care quality and safety.

### Limitations

The study has several limitations, including the variable response rate to each question in the survey. Due to its exploratory nature, the survey was reasonably long (33 questions), potentially affecting participants’ retention as they completed the survey; research has shown that shorter surveys produce higher response rates compared with longer surveys.^28^ We also discovered, after completion, that the settings on 1 survey matrix item were incorrect and thus prevented participants from responding fully to a question (when asked whether centers had a policy in place to support maternal and/or fetal resuscitation, participants could only respond “yes” to either maternal or fetal, not both). Open-ended responses to this item supported the findings, and the results were adjusted according to the individual item response rate; however, this could have limited responses to this item.

## Conclusions

In this global survey of 28 fetal surgical centers, there was little international consensus as to how fetal resuscitation, emergency delivery, and subsequent neonatal resuscitation were managed in practice during fetal surgery for open spina bifida repair. Further collaborative work between centers is required to assess how resuscitation decisions are made and their effect on professionals and parents. To ensure that parents are supported to make informed decisions about their unborn infant undergoing fetal surgery, parental engagement in all aspects of fetal and neonatal resuscitation policy and practice is recommended.

## Notes

Supplement 1. **eAppendix.** Fetal Resuscitation during Fetal Surgery for Spina Bifida Repair: Survey Questions

Supplement 2.
Data Sharing Statement
