# Supplementary material for: Global Policy and Practice for Intrauterine Fetal Resuscitation During Fetal Surgery for Open Spina Bifida Repair
Source: JAMA Netw Open. 2023 Apr 25;6(4):e239855. doi: 10.1001/jamanetworkopen.2023.9855 (PMC10130943; doi:10.1001/jamanetworkopen.2023.9855)
Supplement: Supplement 1. — eAppendix. Fetal Resuscitation during Fetal Surgery for Spina Bifida Repair: Survey Questions [file jamanetwopen-e239855-s001.pdf]

Supplementary Online Content

Gallagher K, Crombag N, Prashar K, et al. Global policy and practice for intrauterine fetal resuscitation during fetal surgery for open spina bifida repair. *JAMA Netw Open.* 2023;6(4):e239855. doi:10.1001/jamanetworkopen.2023.9855

eAppendix. Fetal Resuscitation during Fetal Surgery for Spina Bifida Repair: Survey Questions

This supplementary material has been provided by the authors to give readers additional information about their work.

© 2023 Gallagher K et al. *JAMA Network Open.*

eAppendix.

Fetal Resuscitation during Fetal Surgery for Spina Bifida Repair: Survey Questions

Consent form: Questions 1-8

8. Which surgical centre do you work at? (centre, city, country)

9. Contact email address

10. What is your role at the centre?
(fetal medicine specialist / obstetrician / neurologist / neonatologist / anaesthesiologist / nurse / midwife / other – please specify

11. Does the centre offer open fetal surgery, fetoscopic surgery, or both?
(Response matrix)

12. How many cases of fetal surgery do you undertake, on average, per year?
(Response matrix)

In utero resuscitation
Risks during fetal surgery include critical maternal or fetal haemodynamic compromise, from complications such as circulatory collapse, placental abruption or fetal haemorrhage. In these circumstances, maternal or direct fetal resuscitation measures may be required.

The following questions explore your Centre's experience of maternal or fetal resuscitation during fetal surgery.

13. In the past 5 years, have you had to undertake resuscitation following signs of maternal or fetal compromise (e.g. bradycardia or maternal hypoxia) during open or fetoscopic fetal surgery for spina bifida?
(Response matrix / Resuscitation measures undertaken for the mother and/or the fetus - open ended)

14: Do you currently have a policy in place to support practice in the case of maternal or fetal resuscitation?
(Response matrix / Please upload your maternal and/or fetal resuscitation policy if available - open ended)

15: Who was involved in this policy development?
(N/A / Fetal surgeon / fetal medicine specialist / obstetrician / neurologist / anaesthesiologist / nurse (please specify from where below, i.e. neonatal, surgical) / midwife / patient / patient representatives / other, please share details)

16: Are parents counselled on the potential need for maternal or fetal resuscitation prior to surgery? If yes, which professionals are involved and what is discussed?
(No / Yes – please share details)

© 2023 Gallagher K et al. *JAMA Network Open.*

17: When are parents counselled on the potential need for maternal and/or fetal resuscitation?
(Not counselled / at initial consultation / at consent for surgery / other – please describe below)

18: Do you gain informed signed consent from parents to perform maternal and/or fetal resuscitation measures in the event of maternal or fetal compromise?
(Response matrix)

19: Is the plan for what to do in the event of maternal and/or fetal compromise requiring resuscitation measures discussed with the whole surgical team prior to surgery?
(No / yes – please share details of who does this, when it is discussed, and what is discussed)

Fetal resuscitation during fetal surgery for spina bifida repair
If maternal or fetal resuscitation measures fail to stabilise the maternal and/or fetal circulation, emergent fetal delivery and subsequent neonatal resuscitation may be required. The following questions explore your Centre's experience with emergent fetal delivery during fetal surgery for spina bifida.

Emergent fetal delivery involves the delivery and possible resuscitation of a fetus in the event of critical maternal or fetal compromise not responsive to resuscitation.

20: In the past 5 years, how many cases of emergent fetal delivery during open and/or fetoscopic fetal surgery for spina bifida repair have you had at your Centre?
(Response matrix)

21: If yes to the above, at what gestation was this performed and were neonatal resuscitation measures undertaken? Please include details of why / why not and the outcome of this scenario.
(N/A / yes -please share details)

22: At what gestation would you consider initiating neonatal resuscitation measures following emergent fetal delivery (if the infant shows signs of life)? Please tick all which apply and share your thoughts at the end
(Never / <22 weeks / 22+0-22+6 / 23+0–23+6/24+0-24+6/25+0-25+6/26+0-26+6/27+0-27+6/28+0-28+6/>29+0 weeks / other /please share details or rationale)

23: Do you currently have a policy in place to support practice management in the case of emergent fetal delivery?
(No / yes / please upload policy if available)

24: Who was involved in this policy development?
(N/A / Fetal surgeon / fetal medicine specialist / obstetrician / neurologist / anaesthesiologist / nurse (please specify from where below, i.e. neonatal, surgical) / midwife / patient / patient representatives / other, please share details)

© 2023 Gallagher K et al. *JAMA Network Open.*

25: Are parents counselled on potential emergent fetal delivery and neonatal resuscitation in the event of critical maternal or neonatal compromise not responsive to resuscitation? If yes, which professionals are involved and what is discussed?
(No / yes – please share details)

26: When are parents counselled on potential emergent delivery in the event of critical maternal or fetal compromise not responsive to resuscitation?
(Not counselled / at initial consultation / at consent for surgery / other – please describe)

27: Do you gain informed signed consent from parents to perform emergent fetal delivery and neonatal resuscitation in the event of critical maternal or fetal compromise not responsive to in utero resuscitation?
(Response matrix)

28: Is the plan for what to do in the event of emergent fetal delivery and neonatal resuscitation discussed with the team prior to surgery?
(No / yes – please share details of who does this, when it is done, and what is discussed)

Neonatal Palliative care
Neonatal palliative care describes the provision of support during end-of-life care for the baby and his or her family. The following questions explore how your centre provides neonatal palliative care.

29: Do you discuss a plan for neonatal palliative care in the case of neonatal resuscitation following emergent fetal delivery? If yes, what is discussed in this context?
(No / yes – please share details)

30. Who discusses neonatal palliative care with the parents, and when?
(Open ended)

31: Do you have a policy in place to support practice in the case of fetal death during fetal surgery or following emergent fetal delivery? If so, please describe the support available for families, including information such as when and where parents get to see their baby following surgery.
(No / yes – please share details / please upload relevant policy if available)

32: Are the local palliative care or hospice team involved in end-of-life care / support for families?
(No / yes – please share details)

General comments
33. Please provide any further comments or thoughts around the scenario of fetal resuscitation during fetal surgery

© 2023 Gallagher K et al. *JAMA Network Open.*
