# Supplementary material for: Global Policy and Practice for Intrauterine Fetal Resuscitation During Fetal Surgery for Open Spina Bifida Repair
Source: JAMA Netw Open. 2023 Apr 25;6(4):e239855. doi: 10.1001/jamanetworkopen.2023.9855 (PMC10130943; doi:10.1001/jamanetworkopen.2023.9855)
Supplement: Supplement 2. — Data Sharing Statement [file jamanetwopen-e239855-s002.pdf]

Data Sharing Statement

Gallagher. Global Policy and Practice for Intrauterine Fetal Resuscitation During Fetal Surgery for Open Spina Bifida Repair. *JAMA Netw Open*. Published April 25, 2023.
doi:10.1001/jamanetworkopen.2023.9855

Data
Data available: No
